# Microbial biotechnology for water treatment: An annotated selection of World Wide Web sites relevant to the topics in *microbial biotechnology*

**DOI:** 10.1111/1751-7915.13413

**Published:** 2019-04-15

**Authors:** Lawrence P. Wackett

**Affiliations:** ^1^ Department of Biochemistry, Molecular Biology & Biophysics BioTechnology Institute University of Minnesota St. Paul MN 55108 USA

## Water remediation databases

https://clu-in.org/databases/

This page is put up by the U.S. Environmental Protection Agency. It contains numerous links to information on different remediation projects, involving nanotechnology, phyto‐technology and chemical oxidation.

## Microbial biotechnology for potable water

https://onlinelibrary.wiley.com/doi/full/10.1111/1751-7915.12837

This review is based on the process that biological drinking water treatment processes for removing chemical contaminants are generally less expensive and less energy intensive than other methods.

## Application of biotechnology for water treatment

https://tifac.org.in/index.php/8-publication/154-application-of-biotechnology-for-water-treatment

This site provides a link to a report on biotechnology for advanced water treatment.

## Biological treatment of water: National Academy of Engineering

https://www.nae.edu/7700/BiologicalTreatmentsofDrinkingWater

This treatise on biological water treatment is put out by the U.S. National Academy of Engineering.

## Drylet: Wastewater bioremediation

https://www.drylet.com

Drylet technology combines specific bacteria and porous materials for bioremediation of chemical contaminants in water.

## Minnepura Technologies

http://minnepura.com

Minnepura Technologies focuses on using encapsulated bacteria to remove chemicals from water, using silica‐based materials for the encapsulation.

## ‘Dark matter’ in drinking water treatment plants

https://www.nature.com/articles/srep44350

Dark matter refers to the sequences in waters that are detected in broad‐based genome sequencing and attributed to poorly studied bacterial diversity. This study was devised to help fill gaps in that knowledge.

## Metagenomics in water treatment plant effluents

https://aem.asm.org/content/84/5/e02168-17

This study examines the impact of wastewater treatment effluents on nearby water systems. In this case, discharge into Lake Michigan was investigated.

## Metagenomics in wastewater treatment sludge

https://www.frontiersin.org/articles/10.3389/fmicb.2017.01382/full

This investigation describes the microbial populations found in a pre‐treated raw sludge and a post‐treated dried sludge from wastewater treatment.

## Urban sewage and the antibiotic resistome

https://microbiomejournal.biomedcentral.com/articles/10.1186/s40168-017-0298-y

This paper describes research in which wastewater was sampled from around China and examined for antibiotic resistance genes. Correlations were sought, and found, for resistance genes with other markers.

## Metagenome sequencing of chlorinated water

https://pubs.rsc.org/en/content/articlelanding/2018/ew/c8ew00395e#!divAbstract

There have been many studies of wastewater metagenomes, but this study focused on treated water post‐chlorination, where microbial populations are much lower.

## What's living in your drinking water?

https://microbedetectives.com

This is a colorful commercial website. The company seeks to use metagenomic analysis to advise on enhancing performance for wastewater treatment, biogas production, and increasing drinking water safety.

## DNA sequencing to profile potable water

https://www.wateronline.com/doc/high-throughput-dna-sequencing-to-profile-microbial-water-quality-of-potable-reuse-0001

This webpage provides a good primer on the use of metagenomics for analyzing and improving wastewater treatment.

## Metagenomic analysis of wastewater microbial communities

https://f1000research.com/articles/5-1881/v1

This web article focuses on the ability of microbial communities to biodegrade personal care products, particularly pharmaceuticals.

